# 2353. Distance and Time to Clinic Are Associated with Increased Risk of Detectable HIV-1 Viral Load at a Peripheral Health Center in Rural Western Uganda

**DOI:** 10.1093/ofid/ofac492.160

**Published:** 2022-12-15

**Authors:** Cate Hendren, Ronnie Ndizeye, Nobert Mumbere, Rebecca J Rubinstein, Emma Baguma, Rabbison Muhindo, Varun Goel, Bonnie E Shook-Sa, Moses Ntaro, Mark Siedner, Edgar Mulogo, Ross M Boyce

**Affiliations:** University of North Carolina School of Medicine, Chapel Hill, NC; Department of Community Health, Faculty of Medicine, Mbarara University of Science & Technology, Mbarara, Mbarara, Uganda; Department of Community Health, Faculty of Medicine, Mbarara University of Science & Technology, Mbarara, Mbarara, Uganda; UNC, Chapel Hill, North Carolina; Department of Community Health, Faculty of Medicine, Mbarara University of Science & Technology, Mbarara, Mbarara, Uganda; Department of Community Health, Faculty of Medicine, Mbarara University of Science & Technology, Mbarara, Mbarara, Uganda; Carolina Population Center, University of North Carolina at Chapel Hill, Chapel Hill, North Carolina; Department of Biostatistics, Gillings School of Global Public Health, University of North Carolina at Chapel Hill, Chapel Hill, North Carolina; Department of Community Health, Faculty of Medicine, Mbarara University of Science & Technology, Mbarara, Mbarara, Uganda; Harvard Medical School, Boston, Massachusetts; Department of Community Health, Faculty of Medicine, Mbarara University of Science & Technology, Mbarara, Mbarara, Uganda; University of North Carolina at Chapel Hill, Chapel Hill, North Carolina

## Abstract

**Background:**

Antiretroviral therapy (ART) improves the health of people living with HIV (PLHIV) and reduces HIV transmission. While availability and efficacy of ART have improved in sub-Saharan Africa (SSA), access remains a challenge. Travel burden, measured as travel time, distance, and cost, has been posited as a potential barrier to ART. For example, a previous study at a large, urban referral center in Uganda showed GPS-measured distance was associated with clinic absenteeism. However, others suggest that PLHIV are willing to travel farther for HIV care because of stigma or for higher quality care. Less is known about the effect of travel burden in rural settings where transportation infrastructure is sparse, and there are few transportation options. Therefore, the objective of this study funded by the IDSA GERM Program was to explore potential associations between distance- and time-to-clinic in a highland area of rural western Uganda with HIV outcomes including viral suppression.

**Methods:**

We enrolled 129 adult participants receiving care at the Bugoye ART clinic. Using a handheld GPS device, we mapped routes between participants’ home and clinic recording trip distance, time, and mode of transportation. We abstracted clinical outcomes from participant medical records. Modified Poisson regression with robust error variance was used to estimate risk ratios of associations between main exposures (e.g., distance to clinic, time to clinic) and primary outcomes (e.g., detectable viral load, missed an ART dose, and history of opportunistic infection [OI]).

**Results:**

Distance and time to clinic were significantly and positively associated with probability of detectable viral load. In adjusted analyses, for every 10-km increase in distance, participants were almost twice as likely to have detectable viral loads (PR: 1.95 [95% CI 1.12, 3.41]). For every 10 additional minutes spent traveling, risk of viral non-suppression increased by almost 40% (PR=1.39 [95% CI 1.08, 1.78]). Neither distance nor time to clinic was associated with increased risk having missed an ART dose or history of OI.

**Conclusion:**

These results suggest travel burden may adversely affect achievement of 90/90/90 goals, and novel, decentralized ART distribution mechanisms may be required.

**Disclosures:**

**All Authors**: No reported disclosures.

